# Integrating metabolic profiling of pancreatic juice with transcriptomic analysis of pancreatic cancer tissue identifies distinct clinical subgroups

**DOI:** 10.3389/fonc.2024.1405612

**Published:** 2024-06-26

**Authors:** Alessandra Pulvirenti, Marialuisa Barbagallo, Anna Rita Putignano, Antonio Pea, Rebecca Polidori, Rosie Upstill-Goddard, Nina Cortese, Paolo Kunderfranco, Laura Brunelli, Giulia De Simone, Roberta Pastorelli, Paola Spaggiari, Gennaro Nappo, Nigel B. Jamieson, Alessandro Zerbi, David K. Chang, Giovanni Capretti, Federica Marchesi

**Affiliations:** ^1^ Section of Pancreatic Surgery, Istituto di Ricovero e Cura a Carattere Scientifico (IRCCS) Humanitas Research Hospital, Rozzano, Italy; ^2^ Department of Surgical Oncological and Gastroenterological Sciences (DiSCOG), University of Padua, Padua, Italy; ^3^ Department of Immunology and Inflammation, Istituto di Ricovero e Cura a Carattere Scientifico (IRCCS) Humanitas Research Hospital, Rozzano, Italy; ^4^ Department of General and Pancreatic Surgery-The Pancreas Institute, Verona University Hospital Trust, Verona, Italy; ^5^ Wolfson Wohl Cancer Research Centre, Institute of Cancer Sciences, University of Glasgow, Glasgow, United Kingdom; ^6^ Department of Medical Biotechnology and Translational Medicine, University of Milan, Milan, Italy; ^7^ Bioinformatics Unit, Istituto di Ricovero e Cura a Carattere Scientifico (IRCCS) Humanitas Research Hospital, Rozzano, Italy; ^8^ Laboratory of Metabolites and Proteins in Translational Research, Department of Environmental Health Sciences, Istituto di Ricerche Farmacologiche Mario Negri Istituto di Ricovero e Cura a Carattere Scientifico (IRCCS), Milan, Italy; ^9^ Pathology Department, Istituto di Ricovero e Cura a Carattere Scientifico (IRCCS) Humanitas Research Hospital, Rozzano, Italy; ^10^ Department of Biomedical Sciences, Humanitas University, Pieve Emanuele, Italy; ^11^ West of Scotland Pancreatic Unit, Glasgow Royal Infirmary, Glasgow, United Kingdom

**Keywords:** pancreatic cancer, profiling, metabolism, tumor infiltrating lymphocytes, transcriptomics

## Abstract

**Introduction:**

Metabolic reprogramming is a hallmark feature of pancreatic ductal adenocarcinoma (PDAC). A pancreatic juice (PJ) metabolic signature has been reported to be prognostic of oncological outcome for PDAC. Integration of PJ profiling with transcriptomic and spatial characterization of the tumor microenvironment would help in identifying PDACs with peculiar vulnerabilities.

**Methods:**

We performed a transcriptomic analysis of 26 PDAC samples grouped into 3 metabolic clusters (M_CL) according to their PJ metabolic profile. We analyzed molecular subtypes and transcriptional differences. Validation was performed by multidimensional imaging on tumor slides.

**Results:**

Pancreatic juice metabolic profiling was associated with PDAC transcriptomic molecular subtypes (p=0.004). Tumors identified as M_CL1 exhibited a non-squamous molecular phenotype and demonstrated longer survival. Enrichment analysis revealed the upregulation of immune genes and pathways in M_CL1 samples compared to M_CL2, the group with worse prognosis, a difference confirmed by immunofluorescence on tissue slides. Enrichment analysis of 39 immune signatures by xCell confirmed decreased immune signatures in M_CL2 compared to M_CL1 and allowed a stratification of patients associated with longer survival.

**Discussion:**

PJ metabolic fingerprints reflect PDAC molecular subtypes and the immune microenvironment, confirming PJ as a promising source of biomarkers for personalized therapy.

## 1 Introduction

Pancreatic ductal adenocarcinoma (PDAC) is one of the deadliest cancers, with five-year survival at less than 10% (1). Such poor prognosis is mostly due to the difficulty in diagnosing PDAC at an early stage, as well as to its resistance to anticancer therapies and the lack of biomarkers to predict treatment response. Efforts to improve overall outcomes are focused on the search for new biomarkers and personalized therapeutic approaches, both requiring more granular characterization of the inter-tumor heterogeneity.

Recent development in multi-dimensional analyses, particularly genomic and transcriptomic, have led to the identification of discrete PDAC subgroups (2–10), which classify PDAC into two major transcriptomic subtypes, classical and squamous (basal-like), characterized by distinct gene expression profiles, mutations, and prognosis (4, 7, 10, 11). The squamous subtype is associated with a worse prognosis and is defined by gene programs involved in immuno-suppression and extensive metabolic reprogramming, favoring glycolysis as the primary energy source (4, 7, 8). Conversely, the classical subtype has a more favorable prognosis and commonly presents a transcriptional signature associated with immune infiltrate (immunogenic subtype) and a metabolism based on the oxidation of fatty acids. The impact of molecular subtypes on chemotherapy response is a subject of ongoing investigation in multiple trials (12, 13). Preliminary results from the COMPASS study (NCT02750657) suggest that patients with a classical subtype signature generally respond better to chemotherapeutic treatment (14).

Recent interest has emerged in the correlation between molecular subtypes and metabolic alterations, for the identification of new prognostic markers and the exploration of potential drug-targetable vulnerabilities within tumors (9, 10, 15, 16). In this context, the composition of pancreatic juice (PJ) emerges as a valuable source of tumor metabolic information. We have shown that pancreatic juice of patients having PDAC presents a high level of lactate compared to patients with other pancreatic disorders (17). Moreover, the PJ metabolite composition was heterogeneous among patients, and we could identify metabolic profiles predictive of distinct oncological outcomes, including a metabolic fingerprint prognostic for longer survival. Pancreatic juice heterogeneity might reflect variations transcriptionally determined as well as tumor metabolic characteristics, known to be responsible for many of the alterations occurring in the tumor microenvironment (TME) (18, 19). Immune cells infiltrating cancer tissues are profoundly affected by metabolic alterations and we found an increased density of PD-1^+^ T cells in highly glycolytic tumors (17). To date, pancreatic cancer remains one of the cancers most resistant to immunotherapy, including checkpoint inhibitors (20). A key element of this low responsiveness is suspected to be the low immunogenicity of pancreatic cancer, which could be linked to a poor infiltration of both activated and immunosuppressive T cells (21). On this line, molecular profiles, combined with multi-dimensional analyses of the tumor immune microenvironment, could be exploited to identify actionable alterations to be validated as clinically relevant biomarker signatures.

Given the opportunity of pancreatic juice to serve as a source of biomarkers, we aimed at integrating the PDAC metabolic information, previously achieved through the analysis of the juice (17) with tumor transcriptome and immune profiling. Integrating different hi-resolution analyses (tissue transcriptome, metabolome, and analysis of immune infiltrate) on matched pancreatic biospecimens (tumor tissue and pancreatic juice), may help in identifying PDACs with peculiar tumor vulnerabilities that might respond differently to surgical and anticancer treatments, including immunotherapies.

## 2 Materials and methods

### 2.1 Patients and study design

Twenty-six patients diagnosed with PDAC undergoing pancreatic resection between 2015 and 2017 at Humanitas Research Hospital were included in the study, after signing informed consent. For all patients enrolled, tumor tissue and pancreatic juice were collected at the time of surgery according to protocols approved by the Ethical Committee of the Institution (protocol number n° 595 and 979/20). Patients median age at surgery was 73 years (Min-Max 45–85), 15 (58%) were female. All patients had a tumor in the head of the pancreas with upstream main duct dilatation according to the inclusion criteria. Most of the patients had a Whipple procedure (88,46%), whereas 3 patients underwent total pancreatectomy. One patient (3.8%) received neoadjuvant chemotherapy, following surgery, 13 (50%) patients received adjuvant systemic treatment. On pathology, median tumor size was 3,6 cm (Min-Max 1,4–6,0), 14 (53,84%) patients had R0 resection, 22 (85%) patients had nodal disease and 3 (11,5%) were classified as metastatic for the presence of nodal disease in pericaval lymph nodes (**Supplementary Table S1**). The median follow-up of the study cohort was 38.5 months (Min-Max 7–51) (**Supplementary Figure S1A**). Pancreatic juice was collected intraoperatively and analyzed as previously described (17). Based on the metabolite composition of the juice (17), the cohort of patients had been grouped into 3 metabolic groups (M_CL), from now on referred to as M_CL1 (n=8), M_CL2 (n=9) and M_CL3 (n=9). A second cohort (cohort 2, **Supplementary Table S2**) was included to verify whether patients with PDAC exhibit specific perturbations of metabolites in PJ that differentiate them from patients with other pancreatic/periampullary diseases. This second cohort included 70 patients who underwent pancreatic surgery for PDAC or other diseases between 2015 and 2020 at Humanitas Research Hospital. For all these patients, PJ was collected intraoperatively and analyzed. All patients signed informed consent according to the same protocols approved by the Ethical Committee of the Institution.

### 2.2 RNA sequencing analysis

Transcriptomic analysis was conducted on FFPE PDAC samples using a targeted, ligation-based Templated Oligo Sequencing (TempO-Seq™) as previously described (22). Using the guidance of the H&E slide (one slide/sample, 4 μm thickness), the marked tumor area was excised from the replicate unstained slide, lysed and analyzed using the Human whole transcriptome panel v2.0 (**Supplementary Figure S1B**). In brief, the TempO-Seq assay is based on the annealing of Detector Oligos consisting of a sequence complementary to an mRNA target plus a universal primer binding site, in immediate juxtaposition to each other on the targeted RNA template such that they can be ligated together. Ligated detector oligos were PCR-amplified using a primer set (single-plex PCR reaction, with a single primer pair for each sample), introducing both the adaptors required for sequencing and a sample-specific barcode. The barcode sequences flanking the target sequence were appropriately inserted into the standard Illumina adaptors to permit standard dual-index sequencing of the barcodes and deconvolution of sample-specific reads from the sequencing data using the standard Illumina software. All the PCR-amplified and barcoded samples were pooled into a single library for sequencing. Sequencing reads were demultiplexed using the standard sequencing instrument software for each sample, using the barcodes to give a FASTQ file for each. Read depth, evaluated as average reads/probe, was 465. TempO-Seq sequence files were analyzed using the Tempo-SeqR software package. Each FASTQ file was aligned using the STAR algorithm to a pseudotranscriptome corresponding to the gene panel used in the assay. The resulting raw count matrix was processed in R environment (v4.1.1) (23) using the average of the counts for the genes associated to multiple probes and passed to *DESeqDataSetFromMatrix()* function from DESeq2 (v1.34, RRID: SCR_015687) (24) to be used as input for downstream analyses. Genes expressed in less than 3 samples with normalized counts lower than 5 were filtered out yielding to a total of 16211 genes. Count data were normalized using DESeq2’s median of ratios method by estimating normalization factor for each sample with *estimateSizeFactors()* function prior running *counts()* function with *normalized=TRUE* argument. Regularized log (rlog) transformed counts of the first 500 genes with highest variance were used to perform Principal Components Analysis (PCA) on 26 PDAC samples. Differential expression analysis was performed with *DESeq()* function and for each contrast the resulting significant differentially expressed genes (pvalue < 0.05) with a log2foldchange greater or equal to 1.5 in either direction were highlighted in the Volcano Plots. Differential analysis across 3 clusters was performed by using the Likelihood Ratio Test (LRT). The normalized expression values of up- and down-regulated genes in each sample were used to build a clustered heatmap based on Pearson correlation distance matrix (average linkage) using pheatmap package (RRID: SCR_016418). Furthermore, the lists of the up-regulated genes in M_CL1 compared to, respectively, M_CL2 and M_CL3 clusters were used as input for conducting Gene Ontology over-representation analysis with *enrichGO()* function from clusterProfiler (v4.2.2, RRID: SCR_016884) (25). The enrichment results for the top 10 enriched terms were visualized as dotplot. RNA-seq normalized counts were used to derive immune and stromal cell types in FFPE samples from PDAC patients by running the xCell pipeline (*xCellAnalysis()* R function) (26). xCell scores of selected immune cell types and Immune (combined score of immune cell types) and microenvironment scores (combined score of immune and stromal cell types) were tested for differential enrichment (Mann-Whitney U-test) between each pair of metabolic clusters (M_CL). Patients were grouped in xCell^hi^ and xCell^lo^ by performing hierarchical clustering based on Euclidean distance with complete linkage. Signatures identifying each cluster were designed by using the intersections of the differential expression analysis results (**Supplementary File 1**). Survival analysis of TCGA human PDAC dataset based on the expression of M_CL1 M_CL2 M_CL3 gene signatures was performed using GEPIA2 (http://gepia2.cancer-pku.cn/). Median value of gene expression values was used as group cutoff.

### 2.3 Molecular profiling

Gene set enrichment was performed using the R package ‘GSVA’ (function gsva - arguments: method = “gsva”, mx.diff = TRUE) (27). GSVA implements a non-parametric unsupervised method of gene set enrichment that allows an assessment of the relative enrichment of a selected pathway across the sample space. The output of GSVA is a gene-set by sample matrix of GSVA enrichment scores that are approximately normally distributed. GSVA was used to obtain a score for the classical genes and a score for the squamous genes, as previously described in the ICGC landmark study of pancreas cancer (4). If the squamous score > classical score the sample was ‘Squamous’, if the classical score > squamous score the sample was ‘Classical’ and if the difference between these scores was < 0.3, tumor samples were classified as ‘Intermediate’.

### 2.4 Multiplex immunofluorescence

For each metabolic group (n=2 samples each group), a 5μm slide consecutive to the one used for the transcriptomic analysis was stained with antibodies directed to tumor cells (pan cytokeratin, PCK), T cell populations (CD8, CD4 and FoxP3), macrophages (CD68) and dendritic cells (CD11c). We considered a fragment of 36mm^2^ for each slide and analyzed the whole slide.

### 2.5 Imaging analysis

The computational image analysis was performed by following the Phenocycler Pipeline proposed by Akoya Bioscences. Cell segmentation and phenotyping were performed with the QuPath (28) software by a stepwise approach. Manually defined tumor regions were divided into rectangular tiles of 1500x1500 µm (about 15 tiles/sample) to reduce the computational load. Cell segmentation was performed with the pre-trained deep-learning model StarDist (29). Cell phenotyping was done in a supervised manner by training a cell classifier based on the manual annotation of PCK+, CD68+, CD8+, FOXP3+, CD4+ and CD11+ cells based on specific staining, adopting the Training Object Classifier tool embedded in QuPath and an Artificial Neural Network-based algorithm. Specifically, we annotated from 25 to 30 cells for each phenotype of interest in one sample as a training set. The accuracy of the classification was visually confirmed by an expert pathologist. For each sample, we extracted the frequency of each cell population and the distance between them. The spatial analysis software CytoMAP (v.1.4.21) (30) was used to compute the Pearson Correlation matrix for each sample and investigate which cell types correlated or avoided each other. To elucidate the role of the surrounding pancreatic fibrosis on the PJ findings, we analyzed digitalized FFPE slides of the pancreatic neck resection margin. We generated a random forest classifier for each H&E slide, using tissue annotation and intensity feature extraction to differentiate between normal pancreatic parenchyma, stromal component, and adipose tissue in each case. To enhance the accuracy of the algorithm, smoothed features at 25 and 50 μm radii were incorporated and multiple rounds of tissue classification review were performed.

### 2.6 Untargeted metabolomics profiling

Flow Injection Analysis High-resolution mass spectrometry (FIA-HRMS) was used for untargeted metabolomics profiling of 70 pancreatic juices (12 non PDAC, 58 PDAC) (31). Juice metabolites from each subject were extracted using cold MeOH (1:4), incubated 20 minutes at -80°C, centrifuged at 13000 g for 15 min. A portion of the metabolites extract (8 μL) was analyzed by -Orbitrap QExactive Mass Spectrometer (ThermoFisher Scientific, Waltham, MA, USA) equipped with an electrospray source operated in negative and positive modes. Each run was carried out by injecting 8 μL of sample extract at a flow rate of 50 μL/min (Agilent 1200 Series) of mobile phase consisting of isopropanol/water (60:40, v/v) buffered with 5 mM ammonium at pH 9 for negative mode and methanol/water (60:40, v/v) with 0.1% formic acid at pH 3 for positive mode. Reference masses for internal calibration were used in continuous infusion during the analysis (m/z 210.1285 for positive and m/z 212.0750 for negative ionization). Mass spectra were recorded from m/z 50 to 1000 with 60 000 resolutions. The source temperature was set to 240°C with 25 L/min drying gas and a nebulizer pressure of 35 psig. MS/MS fragmentation pattern of the significant features was collected and used to confirm metabolite identity. All data processing and analysis were done with Matlab R2016a (The Mathworks, Natick, MA) using our in-house developed script (32). Web interface of MetaboAnalyst (www.metaboanalyst.ca) was used to compute multivariate analysis (OPLS-DA).

### 2.7 Statistical analysis

Disease characteristics were summarized using median and range for continuous variables, and frequency and percentages for categorical variables. Differences between metabolic cluster were estimated by Fisher’s exact test and Kruskal-Wallis rank sum test, as appropriate (R software, package: gtsummary). Overall survival was calculated from the date of surgery until the date of death or last follow-up, estimated using Kaplan-Meier methods and compared between subgroups using log-rank test (R software, packages: ggplot2, survminer). A Cox proportional-hazards model was used to study the association between possible risk factors and OS, multiple comparisons were adjusted using the Bonferroni method (R software, package: gtsummary). All P values were based on 2-tailed statistical analysis, and P < 0.05 was considered statistically significant. All analyses were done using R software (R Core Team, Vienna, Austria), and all software packages used were publicly available.

## 3 Results

### 3.1 Integration of metabolic and transcriptomic analysis of pancreatic cancer biospecimens

**Figure 1:**
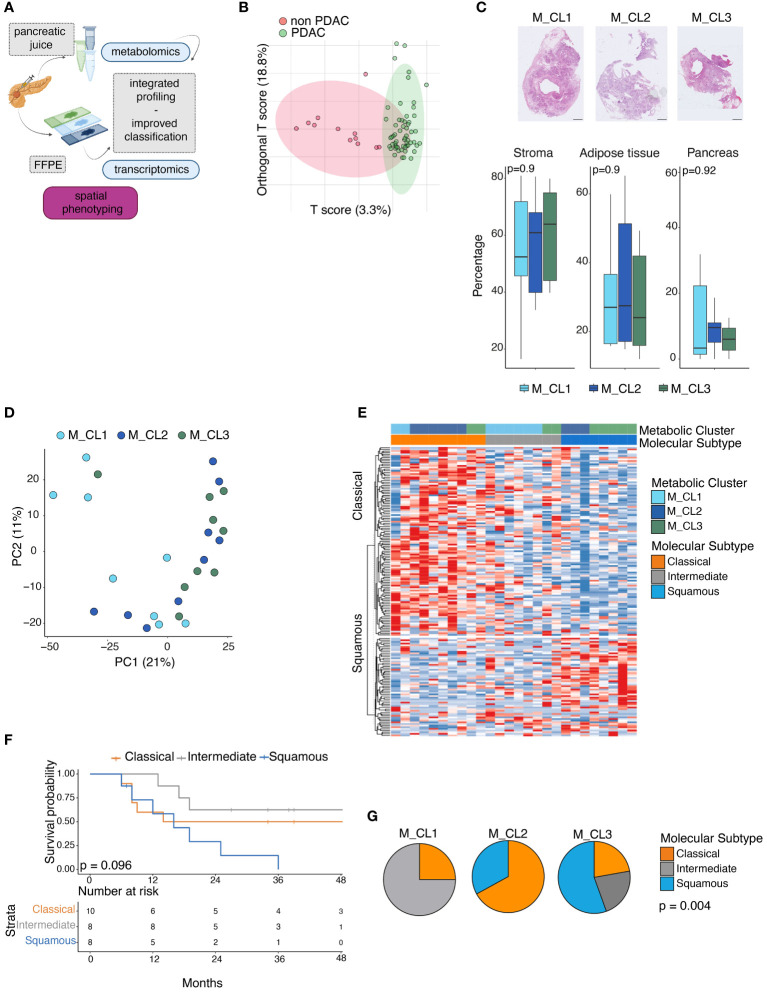
Metabolic and transcriptomic analysis of PDAC paired biospecimens. **(A)** Schematic of the work. Transcriptomic analysis of 26 PDAC sections, on whose pancreatic juice metabolomic analysis had been performed. Integration of multiomics information can improve patient profiling. (*performed with Biorender.com*) **(B)** OPLS-DA score plot obtained from the metabolic profiling of 70 pancreatic juices (12 non PDAC, 58 PDAC). **(C)** Percentage of stromal tissue, adipose tissue and pancreatic glands at the surgical resection margin in the three metabolic groups. Representative pictures of Hematoxylin and Eosin (H&E) staining on tissue slides from M_CL1, M_CL2, M_CL3 residual pancreatic tissue (up). Scale bar: 2mm. Quantification of the three tissue regions by imaging analysis in the three groups (bottom). P=ns by t test. **(D)** Principal component analysis projection of 26 PDAC samples colored by Metabolic cluster assignment. **(E)** Gene set variation analysis (GSVA) of classical and squamous genes, in 26 PDAC samples, according to the Moffitt molecular subtyping. Heatmap showing enrichment scores across 26 samples, color-coded by metabolic cluster and molecular subtype. Samples with score difference lower than 3 are classified as intermediate. **(F)** Survival analysis of patients classified according to the molecular profile. P=0.096 by log-rank test. **(G)** Pie charts showing proportions of molecular subtypes (classical, intermediate and squamous) in the metabolic groups. p= 0.004 by Kruskal-Wallis rank sum test.

To integrate information obtained from paired biospecimens, we performed a transcriptomic analysis on the tumor slide from 26 patients, from whom we had investigated the metabolome of the pancreatic juice collected intraoperatively (17) (**Figure 1A**). Median follow-up among survivors was 38 months (95% CI 8–30 months) (**Supplementary Figure S1A**). Based on the metabolite composition of the juice (17), the cohort of patients had been grouped into 3 metabolic groups (M_CL), from now on referred to as M_CL1 (n=8), M_CL2 (n=9) and M_CL3 (n=9). Patients of the 3 metabolic profiles presented no significant differences in terms of clinical staging parameters such as tumor size [median tumor size M_CL1 (2.8 cm), M_CL2 (3.8 cm) and M_CL3 (4.4 cm)], stage and R status (**Supplementary Table S1**). Nonetheless, at 48 months, the three groups conserved distinct clinical outcomes, particularly highlighting a metabolic profile associated with a favorable prognosis (M_CL1, in which median survival was not reached) and two metabolic profiles characteristics of patients with worse survival, i.e. M_CL2, with a median survival less than one year and M_CL3, with an intermediate outcome (log-rank test p=0.0013) (**Supplementary Figure S1C**). On univariable Cox regression analysis, the metabolic group (M_CL) was the only predictor of survival (Ref: M_CL1, M_CL 2 HR 10.9 95%CI 2.23–53.5, p=0.045, **Supplementary Tables S1**, **S3**). This updated analysis confirmed a correlation between metabolite composition in the juice and survival, extending prognostic insights beyond conventional parameters such as stage and tumor size. Furthermore, in a distinct second cohort (cohort 2, n=70) comparing PDAC with non-PDAC samples, the diagnostic value of the metabolite content of the juice was confirmed by a mass spectrometry-based metabolomic approach (see **Supplementary Methods)** in which the OPLS-DA (orthogonal partial least squares discriminant analysis) score plot showed segregation between PDAC and non-PDAC juices (**Figure 1B**). Lactate and valine were among the metabolites that contributed to this difference, confirming previous findings (17) (**Supplementary Figure S2**).

To exclude that histopathological features (such as the presence of pancreatic fibrosis due to the neoplastic obstructive chronic pancreatitis or atrophy) could have contributed to differences in the pancreatic juice metabolic composition, we analyzed the upstream pancreatic tissue (**Supplementary Figure S1D**) using digital pathology tools. This analysis showed that the proportions of fibrosis, adipose tissue, and pancreatic glands at the surgical resection margin were consistent across the metabolic groups, confirming the absence of correlation between histological characteristics of the surrounding gland and the metabolic signature (**Figure 1C**).

On the same cohort of 26 patients, we performed a transcriptomic analysis of the paraffin-embedded tissue slide from the corresponding primary tumor. We used the TempO-Seq technology (22), a targeted sequencing-based RNA expression analysis, and we deployed to an excised tumor area identified with the guidance of an H&E slide, thus allowing us to include samples with low-tumor content (**Supplementary Figure S1B**), which has represented an issue in previous transcriptomic studies using bulk tissue analysis (33). At PCA visualization there was no clear segregation of the samples previously classified according to their metabolome (M_CL1, M_CL2, M_CL3), suggesting a higher level of complexity of their transcriptome (**Figure 1D**). We then used the transcriptional profile to classify the patient tumor samples as classical or squamous (basal-like), according to the Moffitt molecular subtyping (3–7, 9, 10). Of the 26 samples 10 (38%) resulted in classical and 8 (31%) resulted in the squamous subtype, while 8 (31%) samples presented an intermediate/mixed transcriptome (**Figure 1E**). In line with what was already reported, patients with a squamous molecular subtype had shorter OS (log-rank test, p=0.096) (**Figure 1F**). Integrating the metabolic information with the molecular subtypes, M_CL1, which had the best prognosis presented a higher frequency of intermediate [n = 6 (75%)] and classical [n = 2 (25%)] subtypes with no squamous subtype observed. Conversely, M_CL2, having the worst survival, had a higher frequency of squamous [n = 3 (33%)] and no intermediate subtype; M_CL3 had a mixture of all three subtypes. Differences in subtype distribution among different metabolic clusters were statistically significant (p= 0.004 by Fisher’s exact test) (**Figure 1G**), suggesting a consistency of the molecular subtypes and metabolic profiles.

### 3.2 Transcriptional differences of PDAC tissues between metabolic clusters identify immunologically distinct profiles

**Figure 2:**
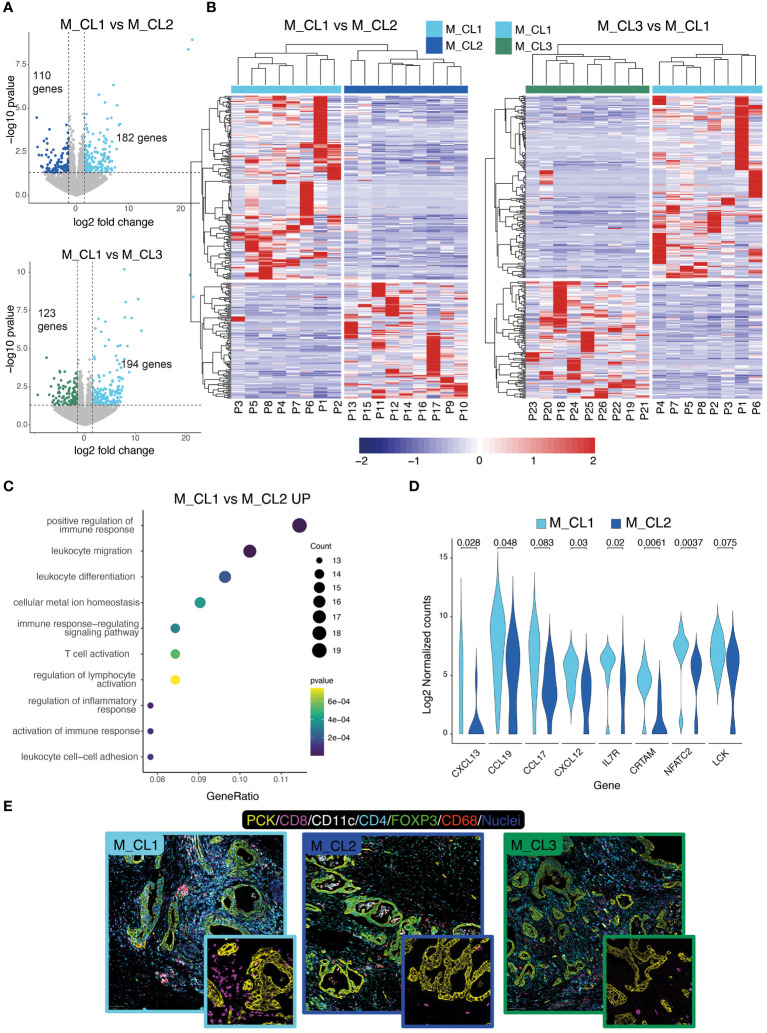
Transcriptional differences of metabolically distinct PDAC tissues. **(A)** Differential gene expression analysis on M_CL1 and M_CL2 (**top**) and M_CL1 and M_CL3 (**bottom**) transcriptomes. Volcano plots of differentially expressed genes (DEGs) highlighting genes having log2(fold change)>|1.5 and p-value<0.05). **(B)** Scaled normalized expression values of upregulated and downregulated DEGs in M_CL1 versus M_CL2 samples (right panel) and M_CL1 versus M_CL3 samples (left panel). Gene expression is color-coded from blue (lower) to red (higher). Columns represent samples, rows represent genes. The color codes in the upper part of the heatmap indicate the metabolic group identity of each sample. Hierarchical clustering is shown as dendrogram over the columns. **(C)** Enrichment pathway analysis of M_CL1 versus M_CL2 samples. Dot-plot shows top 10 enriched GO terms in M_CL1, ordered by gene ratio (percentage of DEGs in the GO term) and color-coded by p-value. The size of the dot represents the count of the genes in the GO term. **(D)** Violin plot showing the log2 normalized counts of selected genes from the T-cell pathways in M_CL1 and M_CL2 samples (p-value by Mann-Whitney U-test). **(E)** Immune contexture in metabolically distinct samples. Representative pictures of M_CL1, M_CL2 and M_CL3 samples stained by multiplex immunofluorescence with antibodies directed against Pan-cytokeratin (yellow), CD8 (magenta), CD4 (cyan), CD11c (white), CD68 (red), FoxP3 (green), nuclei (blue). Scale bar= 100 μm. Insets show CD8+ T cells (magenta) and PCK^+^ tumor cells (yellow).

We then compared the transcriptome of patients belonging to M_CL1, the metabolic cluster with the most favorable prognosis, with the transcriptome of M_CL2 and M_CL3. Differential gene expression (DEG) analysis yielded 182 upregulated genes (M_CL1 compared to M_CL2) (**Figure 2A**, top) and 194 upregulated genes when compared to M_CL3 (**Figure 2A**, bottom). Based on the differentially expressed genes, all the samples clustered according to the metabolic group (**Figure 2B**), confirming that the variations detected in the juice correlate with the transcriptome of the corresponding tumor tissue. Among the top 10 pathways enriched in M_CL1 compared to M_CL2 samples by gene ontology enrichment analysis, many immune pathways emerged, including positive regulation of the immune response, leukocyte migration and T-cell activation (**Figure 2C**), consistent with the up-regulation of genes encoding for lymphoid chemokines (such as *CXCL13*, *CCL19*, *CXCL12*) and genes related to T-cell biology (*CRTAM*, *IL7R* and *NFATC2*) (**Figure 2D**). The comparison with M_CL3 evidenced tissue-specific processes, such as regulation of secretion and response to hormones (**Supplementary Figure S1E**). The transcriptional differences corresponded to a modified tumor microenvironment as assessed by multiplex immunofluorescence on FFPE slides (**Figure 2E**), as immune cells, particularly CD8^+^ and CD4^+^ T cells, infiltrated PCK^+^ tumor tissue considerably in M_CL1, while very few were present in M_CL2 samples (**Figure 2E**). Notably, differential analysis across the 3 groups overlapped with the comparison between M_CL1 and M_CL2.

### 3.3 Spatial assessment of metabolically distinct PDAC tissues

**Figure 3:**
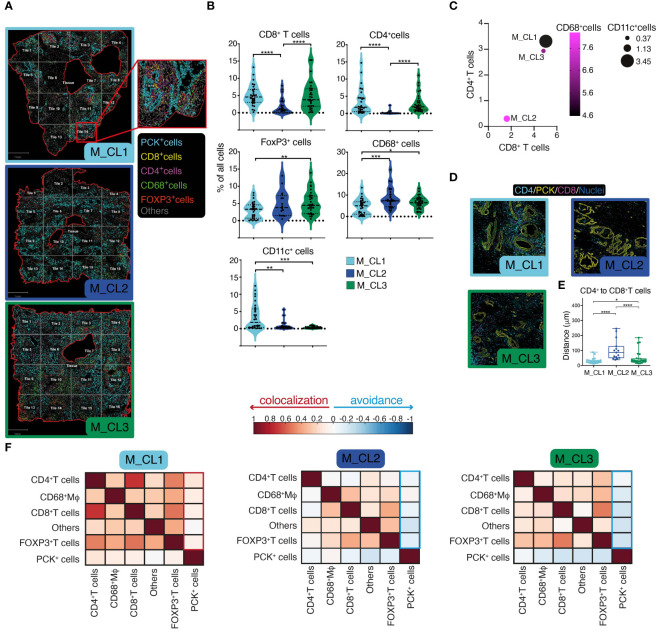
Spatial assessment of PDAC tissues identifies immunologically distinct profiles. **(A)** Multiparametric immunofluorescence on whole slides of PDAC sections, stained for Pan-cytokeratin (yellow), CD8 (magenta), CD4 (cyan), CD11c (white), CD68 (red), FoxP3 (green). Nuclei in (blue). One representative image for each metabolic group (M_CL1, M_CL2 and M_CL3), showing borders of the tissue analyzed (red line), segmentation of the tissue in quadrants (tiles, grey line) and cell phenotyping (inset). Scale bar= 1mm. **(B)** Quantification of main populations in the three groups. Violin plot represents percentage of cells in each tile, dot-lines are first and third quartiles, dashed line represents the median. n=2 samples each group, n=14 tiles in M_CL1 and n=16 tiles in M_CL2 and M_CL3. P value by student t test. **(C)**. Bubble plot showing percentage of CD68^+^ Mφ (circle color), CD11c^+^ DCs (circle size), CD8^+^ T cells (position on X) and of CD4^+^ T cells (position on Y). **(D)** Spatial analysis of the distance between CD8^+^ and CD4^+^ T cells in PDAC tissues. Representative pictures of sections from M_CL1, M_CL2 and M_CL3 samples. CD8 (magenta), CD4 (cyan), PCK+ tumor cells (yellow). Scale bar= 100 μm. **(E)** Quantification of the distance between CD8^+^ and CD4^+^ T cells in PDAC tissues. Dots represent distance in μm between each CD8^+^ T cell and CD4^+^ T cells in each tile. n=2 whole slides each metabolic group, n=14 tiles in M_CL1 and n=16 tiles in M_CL2 and M_CL3. **(F)** Pearson’s correlation coefficients between cells, allowing to understand the colocalization rather than avoidance among different cell types. * p<0.05; ** p<0.01; ***p<0.001; ****p<0.0001.

Having shown a deep-level classification of PDAC tissues, by a metabolic classification based on the PJ and a molecular and immune classification based on the transcriptome, we aimed at integrating this information by quantitative multiparametric immunofluorescence on whole slides (**Figure 3A**). This analysis would indeed contribute to a better understanding of immune cell infiltration in the tumor microenvironment (TME), elucidating their spatial relationships and potential interactions between cells. A significant higher CD8^+^ and CD4^+^ T cell density in M_CL1 and M_CL3 compared to M_CL2 samples emerged (**Figure 3B**), and a lower density in FOXP3^+^ T cells and CD68^+^ macrophages in M_CL1 (**Figure 3B**). Of note CD11c^+^ dendritic cells were almost absent in M_CL2 and M_CL3 samples, compared to M_CL1. These differences were better evidenced when considering all the immune variables together (**Figure 3C**), suggesting that a general adaptive immune infiltration, rather than single populations, was characteristic of M_CL1, the group with the better survival. Spatial analysis of the distance among different immune cells evidenced that CD8^+^ T cells and CD4^+^ T cells were significantly closer in M_CL1 samples, compared to the other 2 groups (**Figures 3D, E**). Moreover, Pearson’s correlation coefficients between cells, evidenced colocalization of all the immune types with PCK^+^ tumor cells in M_CL1 while negative coefficients showed avoidance in M_CL2 and M_CL3 samples (**Figure 3F**).

### 3.4 Transcriptional immune profiles with prognostic significance in PDAC

**Figure 4:**
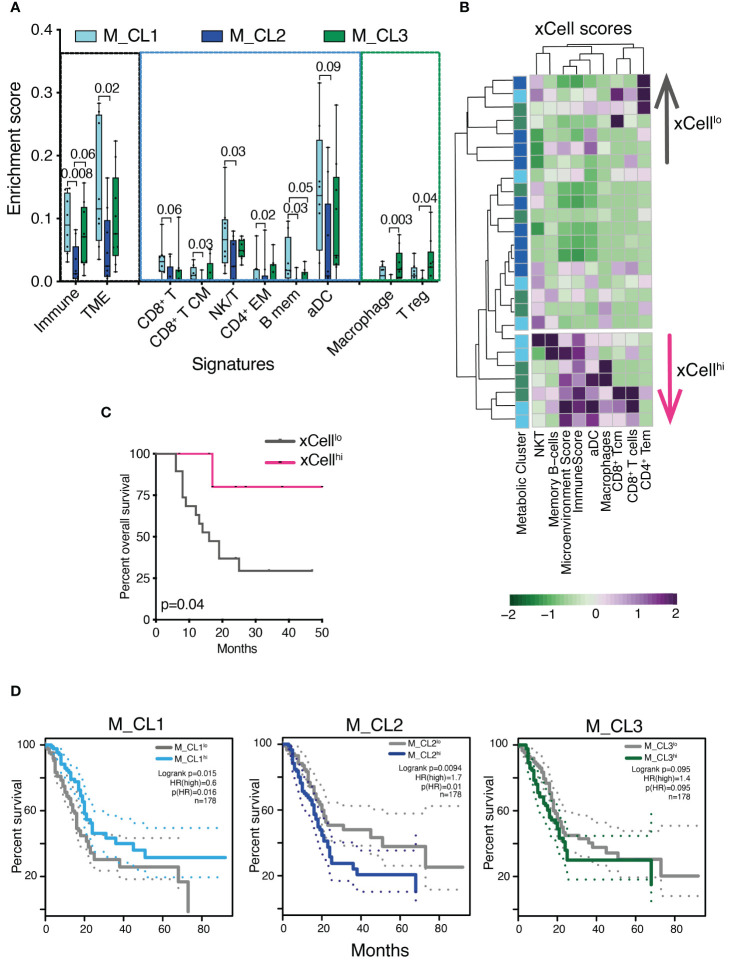
Transcriptional immune profiles are prognostically significant in PDAC. **(A)** Enrichment score of stromal and immune cell types in distinct metabolic groups using the xCell tool. Boxplots show xCell types significantly differentially enriched in M_CL1 (n=8), M_CL2 (n=9) and M_CL3 (n=9) PDAC samples (Mann Whitney U test). Box plots give median, lower and upper quartile by the box and minimum and maximum by the whiskers. **(B)** Heatmap showing xCell scores across 26 PDAC samples, color-coded by metabolic cluster and divided into two groups (xCell^hi^ and xCell^lo^) based on the hierarchical clustering results. Columns represent samples, rows represent cell types. xCell score is color-coded from green (lower) to violet (higher). **(C)** Survival analysis of patients stratified into 2 groups, according to the xCell enrichment score (xCell^hi^ and xCell^lo^). p=0.04 by log rank test. **(D)** Overall survival curves on TCGA human PDAC data based on median expression of gene signatures from M_CL1, M_CL2 and M_CL3. Dotted lines show 95% CI. HR, hazard ratio.

Immune genes and pathways have been shown to contribute to the classification of PDAC into clinically relevant profiles (6). We took advantage of xCell, a signature-based tool that performs enrichment analysis based on gene expression data, to further test whether the transcriptome encoded clinically relevant information. We considered 39 immune signatures, inclusive of main immune types (T, B, NK cells, myeloid cells, stromal and immune signatures) and we computed the signature enrichment score for each sample of our cohort (**Figure 4A**, only significant signatures are shown). The analysis confirmed that samples belonging to M_CL2, the metabolic group with the worst prognosis, presented a marked decrease in the overall immune and microenvironment signatures (**Figure 4A**), in line with the low-grade infiltration of these tumors (**Figure 2E**). Both the T, B and NK cell compartments, alongside activated DC signatures, accounted for the decreased enrichment in M_CL2 compared to M_CL1, while M_CL3 were less homogeneous. This group instead, presented a significant enrichment in macrophage and T-regulatory signatures (**Figure 4A**). Of note, the stratification of patients into 2 groups, according to the xCell enrichment score (xCell^hi^ and xCell^lo^) was significantly associated with longer survival (**Figures 4B, C**). TCGA survival analysis on PDAC indicated a significant correlation between high expression of M_CL1 signature and favorable prognosis (p=0.015 by LogRank). The opposite was observed for M_CL2 and M_CL3 signatures, which correlated with a negative clinical outcome (p=0.0094 and 0.095 by LogRank, respectively) (**Figure 4D**).

## 4 Discussion

Pancreatic ductal adenocarcinoma (PDAC) is characterized by extensive metabolic reprogramming, which is specific to distinct molecular subtypes and has a profound impact on the anti-tumor immune response (4, 8, 18, 34).

Our previous research has suggested a link between the composition of pancreatic juice (PJ) and the metabolic rearrangements occurring in PDAC tumors. Specifically, we found that the metabolic profile of PJ from PDAC patients is enriched in lactate, which mirrors the tumor-increased glycolytic activity. Furthermore, PJ metabolic signatures identified in PJ are predictive of clinical outcomes, including prolonged survival (17). Building upon this, here we combined analyses of paired PJ and PDAC biospecimens to provide a comprehensive understanding of the relationship between PJ metabolic composition, PDAC molecular subtypes, and the immune tumor contexture.

By molecular analysis of the PDAC transcripts, the cohort was classified into classical and squamous (basal-like) samples, the two molecular profiles on which most of the transcriptional analyses have converged so far (7). About one-third of tumor samples presented an intermediate or mixed profile, which is in line with a non-binary molecular classification. In fact, despite the existence of distinct transcriptional subtypes of PDAC being documented (3–7, 9, 10), high-resolution analyses have shown that some PDAC tumor tissues consist of a mixture of classical and squamous cells (35, 36). These mixed subtypes also showed intermediate survival (33, 37). The analysis of PJ metabolic signature in the light of PDAC molecular classification showed that the PJ fingerprint identifying patients with the best prognosis was mostly found in those with intermediate subtypes and classical tumors, with no squamous subtypes observed. Conversely, the PJ signature characterizing patients with worse survival was found in squamous and classical tumors only, with no intermediate molecular subtype tumors. This observation aligns with other molecular classifications of pancreatic cancer that incorporate microenvironmental elements, demonstrating a correlation between metabolic profiles and molecular subtypes. Specifically, glycolytic tumors have been linked with squamous subtype, while lipogenic profiles exhibit correlations with classical subtype ^14^.

Nevertheless, factors beyond molecular subtypes may contribute to the differences in survival among PJ metabolic clusters. The pathway enrichment analysis of tumor transcriptomes has highlighted significant distinctions between PDACs identified by the M_CL1 and M_CL2 signatures. PDACs associated with M_CL1 were enriched in genes related to immune pathways and an increased infiltration of both adaptive and innate immune cells, along with a decrease of T-regulatory cells. Previous studies have demonstrated that a more favorable survival in pancreatic cancer is strongly associated with a high infiltration of CD4^+^ effector and cytotoxic T cells, along with a low level of Tregs, rather than a high total T-cell population (38, 39). In M_CL1 all types of T cells, particularly CD8^+^ and CD4^+^ cells, were closer, which suggests an increased interaction between them. In addition, CD8^+^ cytotoxic T cells were found in proximity to tumor cells, which is thought to favor their antitumor activity (38). Together, these findings suggest that the tumors identified by M_CL1 PJ signature exhibit a highly cytotoxic and immune-rich microenvironment, which is also a hallmark of the classical molecular subtype of PDAC, consistent with our molecular classification showing the absence of squamous tumors in the M_CL1 subtype (4, 40). Ultimately, the stratification of patients according to the tumor xCell enrichment score, which classifies patients according to immune pathway expression was prognostic of survival.

The immune contexture of pancreatic cancer is known to play a critical role in determining the prognosis of PDAC patients (17, 38, 41, 42). Despite deep analyses of PDAC immune contexture in large patient cohorts have helped categorize patients into prognostic groups, the fine immune description achieved has provided limited clinical utility in PDAC management decisions. Improved knowledge of what correlates with the immune contexture (i.e. the metabolic signature of the pancreatic juice) may ultimately leverage its applicability and the introduction of immunotherapeutic approaches. Our study suggests that pancreatic juice holds promise as a tool for identifying potential biomarkers and understanding changes that occur in PDAC, TME, and tumor immune contexture.

Pancreatic juice collection through endoscopic techniques has been demonstrated to offer a viable method for molecular analysis in patients undergoing screening for familial predisposition to pancreatic cancer (43–46). The same techniques could be potentially used for the evaluation of PJ metabolic signatures before surgery or chemotherapy, to test its association with response to treatments. It is therefore crucial to integrate pancreatic juice analysis into future prospective and randomized studies before considering its adoption in clinical practice.

This is the first study integrating metabolic and transcriptomic changes in PJ as a surrogate for pancreatic cancer, however, this investigation is limited by the relatively small, single-institution cohort of patients. Future studies integrating the assessment of metabolic signatures with clinical data on a broader scale are therefore needed. This will allow for the evaluation of preoperative collection safety, validation of the prognostic role of metabolic signatures, as well as the determination of its clinical relevance for treatment selection. In conclusion, our study highlights the potential of PJ as a valuable resource for understanding the metabolic and molecular characteristics of PDAC, as well as its immune tumor contexture. The metabolic signatures identified in PJ exhibit associations with PDAC molecular subtypes, clinical outcomes, and immune pathways, indicating their potential as prognostic biomarkers.

## Data availability statement

The data is deposited on the Zenodo repository, with the accession number: 10.5281/zenodo.11519719.

## Ethics statement

The studies involving humans were approved by Ethical Committee of Humanitas research Hospital (protocol number n° 595 and 979/20). The studies were conducted in accordance with the local legislation and institutional requirements. The participants provided their written informed consent to participate in this study.

## Author contributions

APu: Formal Analysis, Writing – review & editing, Writing – original draft, Investigation, Conceptualization. MB: Investigation, Writing – review & editing, Formal analysis. ARP: Investigation, Writing – review & editing, Formal analysis, Data curation. APe: Writing – review & editing, Investigation, Formal analysis. RPo: Writing – review & editing, Investigation, Formal analysis. RU-G: Writing – review & editing, Formal analysis. NC: Writing – review & editing, Investigation, Formal analysis. PK: Writing – review & editing, Formal analysis, Data curation. LB: Writing – review & editing, Investigation, Formal analysis. GS: Writing – review & editing, Formal analysis. RPa: Writing – review & editing, Formal analysis. PS: Writing – review & editing, Resources. GN: Writing – review & editing, Resources. NJ: Writing – review & editing, Resources. AZ: Writing – review & editing, Resources. DC: Writing – review & editing, Supervision, Resources. GC: Writing – review & editing, Resources, Funding acquisition, Formal analysis. FM: Writing – review & editing, Writing – original draft, Supervision, Investigation, Funding acquisition, Conceptualization.
