# Supplementary material for: Integrating metabolic profiling of pancreatic juice with transcriptomic analysis of pancreatic cancer tissue identifies distinct clinical subgroups
Source: Front Oncol. 2024 Jun 26;14:1405612. doi: 10.3389/fonc.2024.1405612 (PMC11234733; doi:10.3389/fonc.2024.1405612)
Supplement: Supplementary file 1 [file DataSheet_1.pdf]

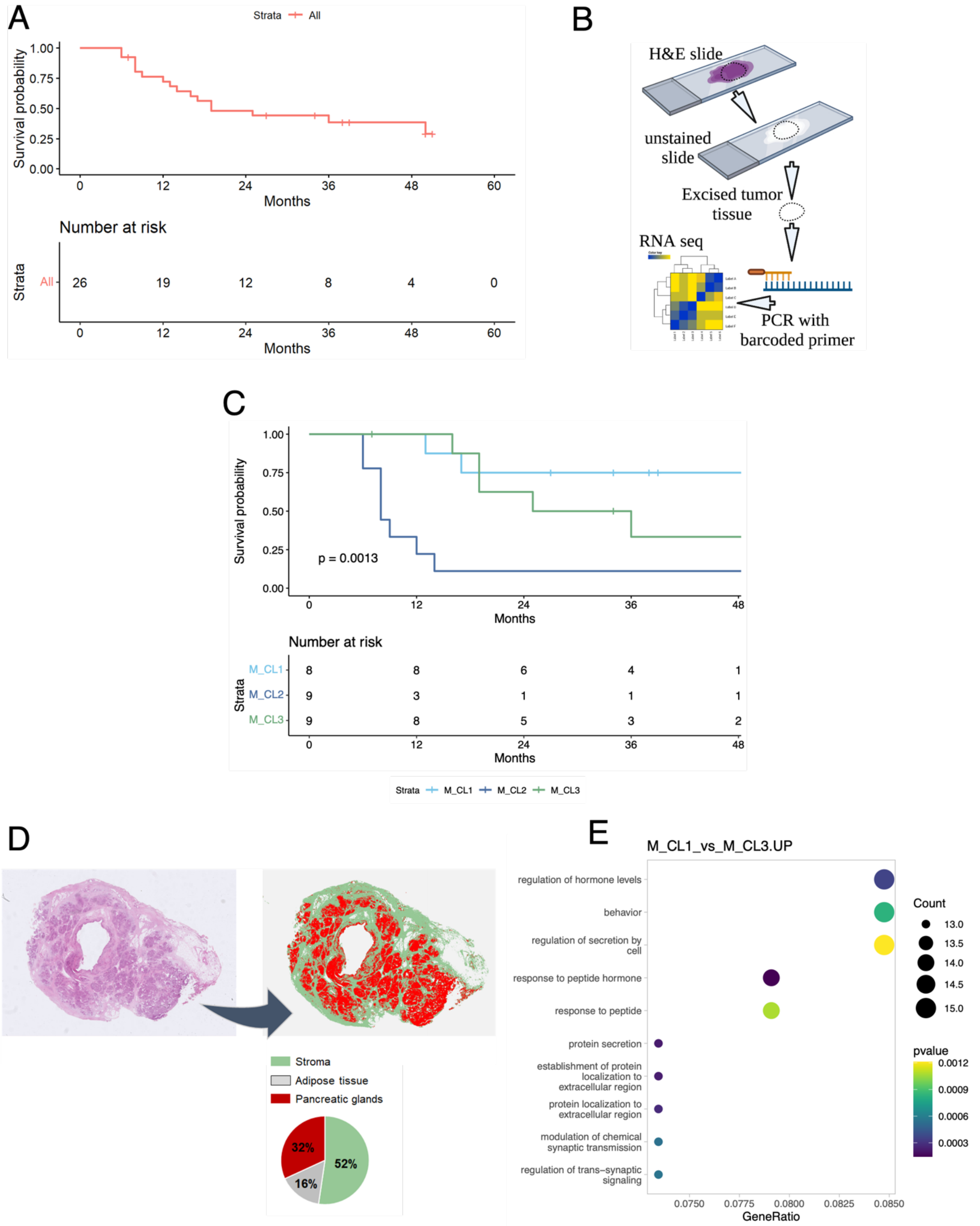
A
Strata All
Survival probability
1.00
0.75
0.50
0.25
0.00
0 12 24 36 48 60
Months
Number at risk
Strata
All 26 19 12 8 4 0
0 12 24 36 48 60
Months
B
H&E slide
unstained slide
Excised tumor tissue
RNA seq
PCR with barcoded primer
C
Survival probability
1.00
0.75
0.50
0.25
0.00
p = 0.0013
0 12 24 36 48
Months
Number at risk
Strata
M_CL1 8 8 6 4 1
M_CL2 9 3 1 1 1
M_CL3 9 8 5 3 2
0 12 24 36 48
Months
Strata M_CL1 M_CL2 M_CL3
D
Stroma
Adipose tissue
Pancreatic glands
32%
52%
16%
E
M_CL1_vs_M_CL3.UP
regulation of hormone levels
behavior
regulation of secretion by cell
response to peptide hormone
response to peptide
protein secretion
establishment of protein localization to extracellular region
protein localization to extracellular region
modulation of chemical synaptic transmission
regulation of trans−synaptic signaling
0.0750 0.0775 0.0800 0.0825 0.0850
GeneRatio
Count
13.0
13.5
14.0
14.5
15.0
pvalue
0.0012
0.0009
0.0006
0.0003

Supplementary Figure 1. (**A**) Overall survival for the whole population cohort. Median OS is 38 months (95% CI 8-30 months) (**B**) Schematic of the TempO-Seq™ workflow. (**C**) Survival analysis of patients categorized as M_CL1, M_CL2, M_CL3 according to metabolic profile (p=0.0013 by log-rank test). (**D**) Schematic of the digital pathology approach used to annotate pancreatic tissue as stroma, adipose or pancreatic gland. (**E**) Enrichment pathway analysis of M_CL1 versus M_CL3 samples. Dot-plot shows top 10 enriched GO terms in M_CL1, ordered by gene ratio (percentage of DEGs in the GO term) and color-coded by p-value. The size of the dot represents the count of the genes in the GO term.

Metabolites	Fold Change PDAC vs non PDAC
**Dihydrothymine**	1.40
**Lactic acid**	1.62
**3-Hydroxymethylglutaric acid**	1.75
**Uridine**	1.99
**Valine**	2.02
**Methionine sulfone**	2.12
**N8-Acetylspermidine**	2.53
**Butyrylcarnitine**	2.96
**Pantothenic acid**	3.03
**Cysteine-S-sulfate**	3.19
**Methionine sulfoxide**	3.49
**Galacturonic acid**	3.60
**Alanylisoleucine**	4.05
**Glycerophosphocholine**	-5.14
**N-Acetyl-L-aspartic acid**	-2.12
**Ethanolamine**	-1.54
**Isopentenyl pyrophosphate**	-1.26
**4-Guanidinobutanoic acid**	-1.17

Supplementary Figure 2. Metabolites significantly different in PDAC versus non PDAC juices (by Wilcoxon-Mann-Whitney test), confirmed by MS/MS.

Variable	N	**Overall**, N = 26[1]	**Cluster 1**, N = 8[1]	**Cluster 2**, N = 9[1]	**Cluster 3**, N = 9[1]	p-value[2]
**Molecular subtype**	26					**0.004**
Classical		10 (38%)	2 (25%)	6 (67%)	2 (22%)	
Intermediate		8 (31%)	6 (75%)	0 (0%)	2 (22%)	
Squamous		8 (31%)	0 (0%)	3 (33%)	5 (56%)	
**Gender**	26					0.2
Female		15 (58%)	5 (62%)	7 (78%)	3 (33%)	
Male		11 (42%)	3 (38%)	2 (22%)	6 (67%)	
**Age at surgery**	26	72.50 (45.00-85.00)	76.50 (45.00-85.00)	72.00 (59.00-78.00)	68.00 (56.00-82.00)	0.3
**BMI**	26	24.00 (20.00-34.00)	23.50 (21.00-27.00)	22.00 (20.00-34.00)	25.00 (23.00-27.00)	0.3
**Diabetes**	26	13 (50%)	3 (38%)	4 (44%)	6 (67%)	0.6
**Neoadjuvant therapy**	26	1 (3.8%)	0 (0%)	1 (11%)	0 (0%)	>0.9
**Adjuvant therapy**	26	13 (50%)	4 (50%)	3 (33%)	6 (67%)	0.4
**Perineural invasion**	26	24 (92%)	7 (88%)	8 (89%)	9 (100%)	0.8
**Lymphovascular invasion**	26	22 (85%)	6 (75%)	8 (89%)	8 (89%)	0.7
**R status**	26					0.092
R0		14 (54%)	7 (88%)	3 (33%)	4 (44%)	
R1		12 (46%)	1 (12%)	6 (67%)	5 (56%)	
**MPD diameter, mm**	19	5.00 (2.00-10.00)	6.00 (2.00-10.00)	5.00 (3.00-7.00)	4.50 (3.00-7.00)	0.8
(Missing)		7	1	3	3	
**T**	26					0.5
T1		4 (15%)	2 (25%)	1 (11%)	1 (11%)	
T2		11 (42%)	4 (50%)	5 (56%)	2 (22%)	
T3		11 (42%)	2 (25%)	3 (33%)	6 (67%)	
**N**	26					0.5
N0		5 (19%)	3 (38%)	1 (11%)	1 (11%)	
N1		9 (35%)	3 (38%)	2 (22%)	4 (44%)	
N2		12 (46%)	2 (25%)	6 (67%)	4 (44%)	
**M**	26					0.3
M0		23 (88%)	6 (75%)	8 (89%)	9 (100%)	
M1		3 (12%)	2 (25%)	1 (11%)	0 (0%)	
**Stage**	26					0.5
I		3 (12%)	1 (12%)	1 (11%)	1 (11%)	
II		10 (38%)	4 (50%)	2 (22%)	4 (44%)	
III		10 (38%)	1 (12%)	5 (56%)	4 (44%)	
IV		3 (12%)	2 (25%)	1 (11%)	0 (0%)	

[1] n (%); Median (Minimum-Maximum)
[2] Fisher's exact test; Kruskal-Wallis rank sum test

Supplementary Table 1. Characteristics of the study population.

**Characteristic**	**N (%)**
**Gender**	
Female	35 (50)
Male	35 (50)
**Age at surgery**	
Median; range	70; 43-85
≤70 yrs/>70 yrs	35 (50)/35 (50)
**Neoadjuvant therapy**	
Yes/No	11 (16)/59 (84)
**Diagnosis**	
Ductal Adenocarcinoma	58 (83)
Pancreatitis	2 (3)
Papillary-Ampulla Tumor	7 (10)
Neuroendocrine	2 (3)
IPMN	1 (1)
**BMI**	
Median; range	23; 16-32
**Perineural invasion***	
Yes/No	58 (83)/10 (15)
**Lymphovascular invasion***	
Yes/No	55 (83)/13 (15)
**R status***	
R0	30 (52)
R1	28 (48)
**Nodal Status***	
N0	14 (24)
N1	19 (33)
N2	25 (43)
**T status***	
T1	4 (7)
T2	33 (57)
T3	20 (35)
**M***	
M0	51 (88)
M1	7 (12)
**Tumor grade***	
G1-2	15 (26)
G3-4	40 (69)

Supplementary Table 2. Demographics and Clinicopathological Characteristics of 70 patients with pancreatic diseases (cohort 2).
