# Supplementary material for: Integrating metabolic profiling of pancreatic juice with transcriptomic analysis of pancreatic cancer tissue identifies distinct clinical subgroups
Source: Front Oncol. 2024 Jun 26;14:1405612. doi: 10.3389/fonc.2024.1405612 (PMC11234733; doi:10.3389/fonc.2024.1405612)
Supplement: Supplementary file 2 [file DataSheet_2.pdf]

Characteristic	Event N	HR[1]	95% CI[1]	p-value	q-value[2]
**Metabolic cluster**					
Cluster 1	2	—	—		
Cluster 2	8	10.9	2.23, 53.5	**0.003**	0.045
Cluster 3	6	2.90	0.58, 14.4	0.2	>0.9
**Tumor diameter, cm**	16	1.02	0.78, 1.33	>0.9	>0.9
**T**					
T1	1	—	—		
T2	7	3.59	0.44, 29.5	0.2	>0.9
T3	8	3.52	0.44, 28.3	0.2	>0.9
**N**					
N0	2	—	—		
N1	4	1.00	0.18, 5.46	>0.9	>0.9
N2	10	2.74	0.60, 12.6	0.2	>0.9
**M**					
M0	14	—	—		
M1	2	1.25	0.28, 5.55	0.8	>0.9
**Stage**					
I	1	—	—		
II	5	1.21	0.14, 10.5	0.9	>0.9
III	8	2.61	0.32, 21.1	0.4	>0.9
IV	2	2.15	0.19, 24.0	0.5	>0.9
**R_status**					
R0	7	—	—		
R1	9	1.92	0.71, 5.19	0.2	>0.9
**Molecular subtype**					
Classical	5	—	—		
Intermediate	4	0.84	0.22, 3.19	0.8	>0.9
Squamous	7	2.68	0.80, 8.93	0.11	>0.9

[1] HR = Hazard Ratio, CI = Confidence Interval

[2] Bonferroni correction for multiple testing

Table S3. Univariate Cox Regression Table.
